# Correction

**DOI:** 10.1080/19420862.2024.2335597

**Published:** 2024-03-28

**Article title**: A pivotal decade for bispecific antibodies?

**Authors**: Marlena Surowka and Christian Klein

**Journal**: *MABS*

**DOI**: https://doi.org/10.1080/19420862.2024.2321635

In the original version of this article, Figures 1 and 2 had errors introduced.

**Figure 1:**
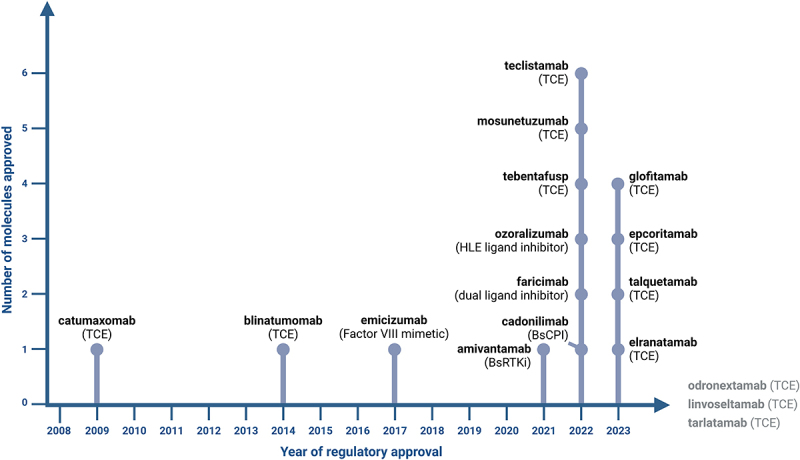

**Figure 2:**
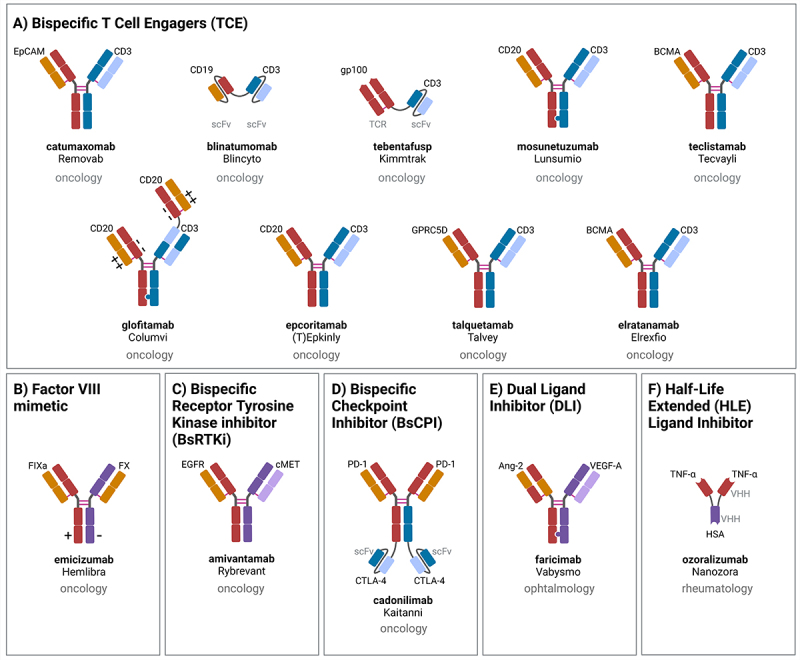

Correct versions of figures are as below.

